# Multi-organ single-cell transcriptomics of immune cells uncovered organ-specific gene expression and functions

**DOI:** 10.1038/s41597-024-03152-z

**Published:** 2024-03-27

**Authors:** Maria Tsagiopoulou, Sonal Rashmi, Sergio Aguilar-Fernandez, Juan Nieto, Ivo G. Gut

**Affiliations:** 1 Centro Nacional de Analisis Genomico (CNAG), Barcelona, Spain; 2 Universitat de Barcelona (UB), Barcelona, Spain

**Keywords:** Data integration, Cell migration, Gene regulation in immune cells, Chemokines

## Abstract

Despite the wealth of publicly available single-cell datasets, our understanding of distinct resident immune cells and their unique features in diverse human organs remains limited. To address this, we compiled a meta-analysis dataset of 114,275 *CD45*+ immune cells sourced from 14 organs in healthy donors. While the transcriptome of immune cells remains relatively consistent across organs, our analysis has unveiled organ-specific gene expression differences (*GTPX3* in kidney, *DNTT* and *ACVR2B* in thymus). These alterations are linked to different transcriptional factor activities and pathways including metabolism. *TNF-α* signaling through the *NFkB* pathway was found in several organs and immune compartments. The presence of distinct expression profiles for *NFkB* family genes and their target genes, including cytokines, underscores their pivotal role in cell positioning. Taken together, immune cells serve a dual role: safeguarding the organs and dynamically adjusting to the intricacies of the host organ environment, thereby actively contributing to its functionality and overall homeostasis.

## Introduction

Immune response is classified into: (i) innate, involving dendritic, macrophages, granulocytes, and NK cells and (ii) adaptive, encompassing B and T cells. The majority of immune cells are produced in the bone marrow and circulate through the bloodstream to various organs. Our understanding of human immune cells is largely derived from immune cells in peripheral blood and there is limited understanding of the diversity, specificity and behavior of immune cells across different organs. State-of-the-art technologies to analyze individual cells such as single-cell RNA-seq (scRNA-seq) have been used to study organ-specific immunity^1,2^.

Public datasets of scRNA-seq are increasing through international collaborative efforts such as the Human Cell Atlas (HCA) charting the cell types in the healthy body, across time from fetal development to adulthood, and eventually to old age^3^. There has been little attempt to integrate the scRNA-seq data from different sources^4^. However, recent publications have successfully mapped cell types across various human tissues, presenting pan-tissue atlases from a different perspective^5–8^. Dominguez Conde *et al*. focused on the immune cell analysis in different tissues and provided an in-depth characterization of a hundred immune cell populations, with a primary emphasis on macrophages and memory T cells.^5^. Despite these advancements, comprehensive multi-organ meta-atlases addressing specific or shared genes and functions of immune cells concerning their host organs remain largely unexplored.

Since the first efforts to characterize the immune profile of human organs started a wealth of data has become publicly available that could be harnessed for integration. In this context, we analyzed the transcriptional landscape of 114,275 immune cells across 14 distinct organs addressing the unique and shared features across different organs. In this study, we aimed to investigate whether immune cells undergo alterations upon residency in a host organ, their utilization of host organ components, and whether they play roles beyond their defensive functions within the host organ. Specifically, we sought to understand the mechanisms underlying immune cell entry into host organs. Our findings reveal noteworthy gene expression changes with a remarkable specificity for particular organs across immune cell types. The altered genes were associated with either specific or shared transcription factor activity and biological pathways, such as the *NFkB* signaling pathway, which is shared across immune cell types and organs, influencing cytokine expression.

## Results

### Multi-organ immune cells map distinct immune cell populations

**Fig. 1:**
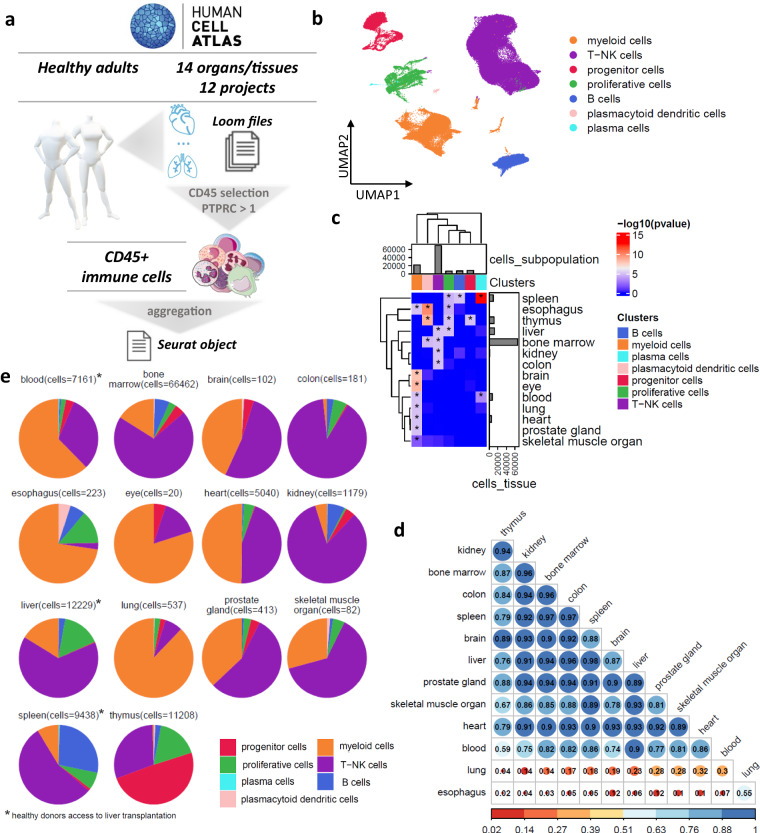
Single-cell transcriptomics of immune cells from different organs reveal the common immune cell subpopulations. (**a**) Graphical representation of the study. (**b**) The UMAP plot of the immune cells. (**c**) Heatmap showing the -log10(p-value) of the hypergeometric distribution between the immune cell subpopulations and the different organs. The asterisks highlight the statistically significant enrichment (p-value < 0.05). (**e**) Pie charts displaying the frequencies of the immune cell subtypes across the different organs. (**d**) Pearson correlation plot across the different organs.

We aggregated 162 healthy donor samples sourced from 14 organs (Supplementary Table 1) and 12 different projects (1/12 from liver transplantation) from the HCA portal (https://data.humancellatlas.org/) focusing on *CD45*+ compartment (Fig. 1a). Following a quality control process, the finalized dataset comprised 114,275 high-quality *CD45*+ cells **(**Supplementary-Fig. 1a). Notably, the *CD45*+ selection led to a reduction in the progenitor lineage due to low expression of *CD45*.

**Fig. 2:**
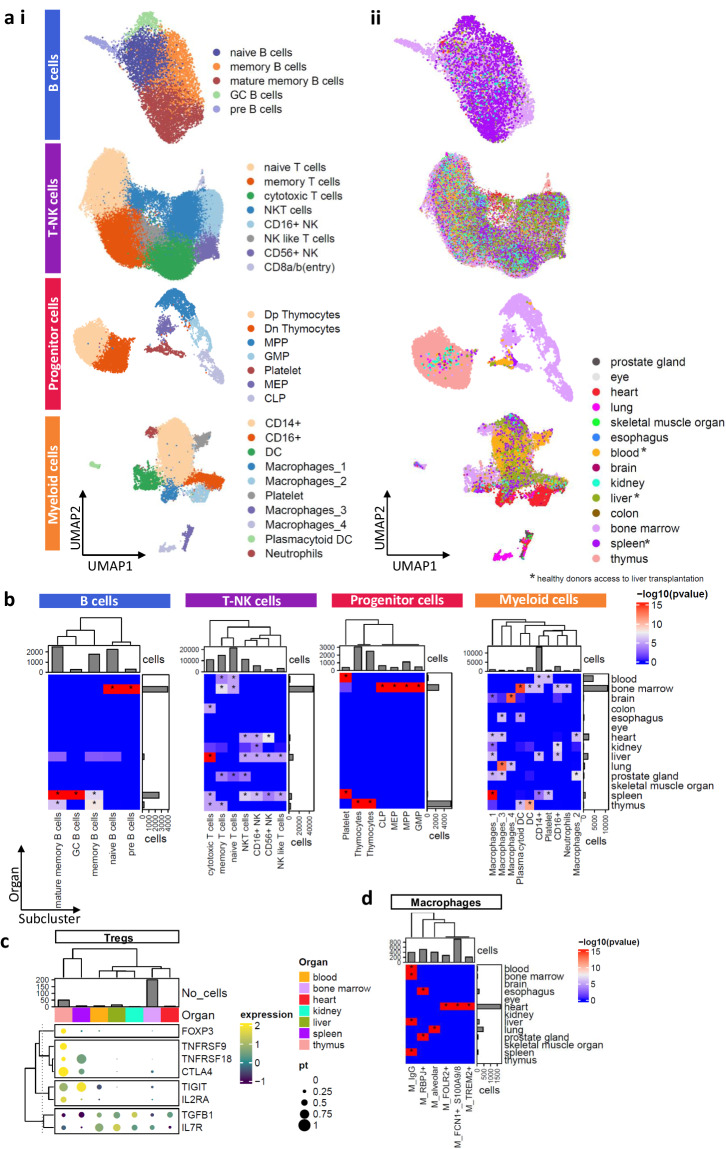
Mapping the different immune cell types across the different organs. (**a**) The UMAP plots of the (i) different immune cell types in (ii) different organs. The asterisks on organs highlighting the liver transplantation. (**b**) Heatmap showing the -log10(p-value) of hypergeometric distribution between the immune cell subpopulations and the different organs. The asterisks highlight the statistically significant enrichment (p-value < 0.05). (**c**) Hierarchical clustering based on expression levels and percentage of cells expressing the Tregs markers. Barplot showing the number of Tregs across organs. (**d**) Heatmap showing the -log10(p-value) of hypergeometric distribution across macrophages and different organs.

We aimed to unravel the heterogeneity of immune cell composition across organs (prostate gland, eye, heart, skeletal muscle organ, blood, liver, brain, kidney, colon, esophagus, and lung) including lymphoid organs (thymus, bone marrow, spleen). We identified the major cell types: T-NK (n = 69,285, 60.6%), myeloid (n = 21,451, 18.8%), B (n = 7,342, 6.4%), plasmacytoid dendritic (n = 349, 0.3%) and plasma (n = 162, 0.1%) cells and two states that thread across cell types: progenitors (n = 9,422, 7.7%) and proliferative cells (n = 6,912, 6%) (Fig. 1b**)**. The clusters did not show a consistent association with cell cycling phases across clusters, except for one proliferative cell cluster, which was excluded from further analysis due to its mixed composition with various immune cell types (Supplementary-Fig. 1b). The organ with the highest representation in terms number of immune cells was the bone marrow (n = 66,465, 58.1%) followed by the liver (n = 12,513, 10.9%), thymus (n = 11,208, 9.8%), and spleen (n = 9,743, 8.5%) (Table 1). Statistical analysis using the hypergeometric distribution (p < 0.05) showed significant association of myeloid cells with several organs, including the brain, eye, blood, lung, prostate gland, heart, esophagus, and skeletal muscle (Fig. 1c–e). Spleen was linked with B cells and together with blood were associated with plasma cells from thymus (hypergeometric distribution, p < 0.05). Proliferative cells showed a connection with the spleen, liver, esophagus and thymus (hypergeometric distribution, p < 0.05). Additionally, bone marrow, liver, kidney, and colon were associated with T cells, with bone marrow showing an additional link to plasmacytoid dendritic cells (DC) thymus (hypergeometric distribution, p < 0.05). To explore immune cell expression profiles across different organs, we employed the Pearson correlation coefficient (r) (Fig. 1d). The results (Pearson’s-r: minimum 0.02-maximum 0.96) showed completely uncorrelated profiles of lung and esophagus compared to the remaining organs with a range of Pearson’s-r 0.04–0.3 and 0.02–0.12, respectively, however only a weak correlation between them was found (Pearson’s-r = 0.55). The best correlation was identified between bone marrow and the colon (Pearson’s-r = 0.96) and kidney (Pearson’s-r = 0.96). A public annotation of the tonsil was used to characterize the immune cells in detail^9^. B and myeloid cells had the same cell-annotation, but the T-NK cells were more finely characterized using this reference (Supplementary-Fig. 2).

**Table 1:** The studied organs and the number of CD45+ immune cells.

Organ	No of cells
bone marrow	66,465
liver	12,513
thymus	11,208
spleen	9,743
blood	7,216
heart	5,040
kidney	1,179
lung	537
prostate gland	414
esophagus	223
colon	181
brain	102
skeletal muscle organ	82
eye	20

### B and progenitor (lymphoid and myeloid lineages) cell analysis revealed thymus properties, thymic B cells and an association with kidney

**Fig. 3:**
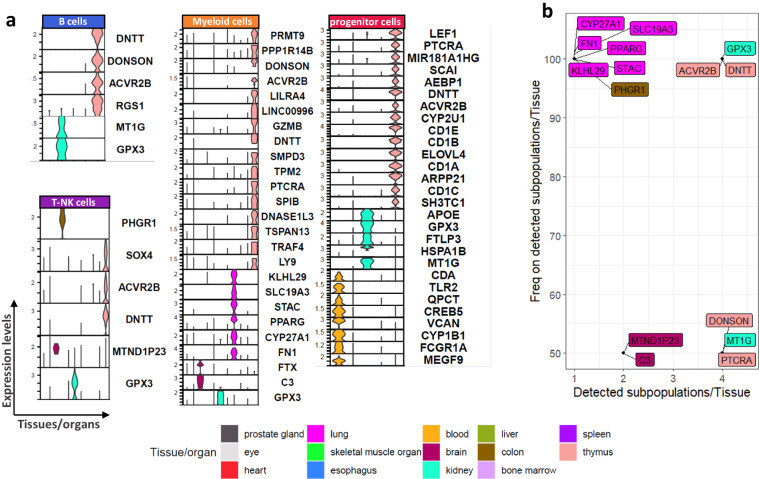
Organ-related signatures per immune subpopulations. (**a**) Violin plots showing the expression of the gene signatures per immune subtype and in each organ. (**b**) Scatterplot showing the frequency of the detected immune cell subpopulations containing the gene in their signatures (x-axis) and the number of the detected immune cell types in each organ (y-axis).

The 7,342 B cells (6.4% of detected immune cells) grouped into five clusters, including one cluster of immature stage expressing *SOX4* (pre B cells) (Fig. 2a, Supplementary-Fig. 3a). The mature memory B cell cluster characterized by *CD27*+ B cells (n = 2,550, 34.7% of detected B cells) combined with memory B cells (n = 1,819) were the overrepresented subpopulations with 59.5% of the defining B cells (Fig. 2b). Of the 7,342 B cells, 3,963 (53.9%) were found in the bone marrow, 2,610 (35.5%) in the spleen, 316 (4.3%) in the liver and interestingly 258 in the thymus. A pre-B cell (n = 312) cluster was organ-specific since the bone marrow plays the main role in the production of B cells (Fig. 2b). Also, bone marrow B cells were associated with naive B cells (n = 1,604, 40.4% of bone marrow B cells) (hypergeometric distribution, p < 0.05). The thymic B cells were linked mainly in mature memory B cells and memory B cells highlighting their critical role in the maturation of T cells^10^. The best correlations were observed between spleen and liver, liver and blood, thymus and kidney (Pearson’s-r: 0.99) (Supplementary-Fig. 3b) while the lowest correlation was between heart, kidney and thymus with liver and spleen. Focused analysis of mature and memory B cells from thymus showed the best correlation with the cells located in bone marrow (Supplementary-Fig. 3c) supporting evidence of thymic B cells origin.

We obtained 9,422 progenitor cells characterized by the expression of classical markers such as *SOX4* **(**Supplementary-Fig. 3d**)**. As expected, the overrepresented organs in this immune subtype were the thymus (n = 5,524, 58.6%) and bone marrow (n = 2,791, 29.6%) (Fig. 2a). The thymus was associated with Dp and Dn thymocytes and bone marrow with myeloid and lymphoid lineage (hypergeometric distribution, p < 0.05) (Fig. 2b). The detected platelet cells (n = 422) were associated with blood and spleen (hypergeometric distribution, p < 0.05). Interestingly, the progenitor cells derived from the kidney were clustered with the thymocytes (46/47 progenitor cells from kidney).

### High diversity of T and myeloid cells and organ specificity of macrophages

**Fig. 4:**
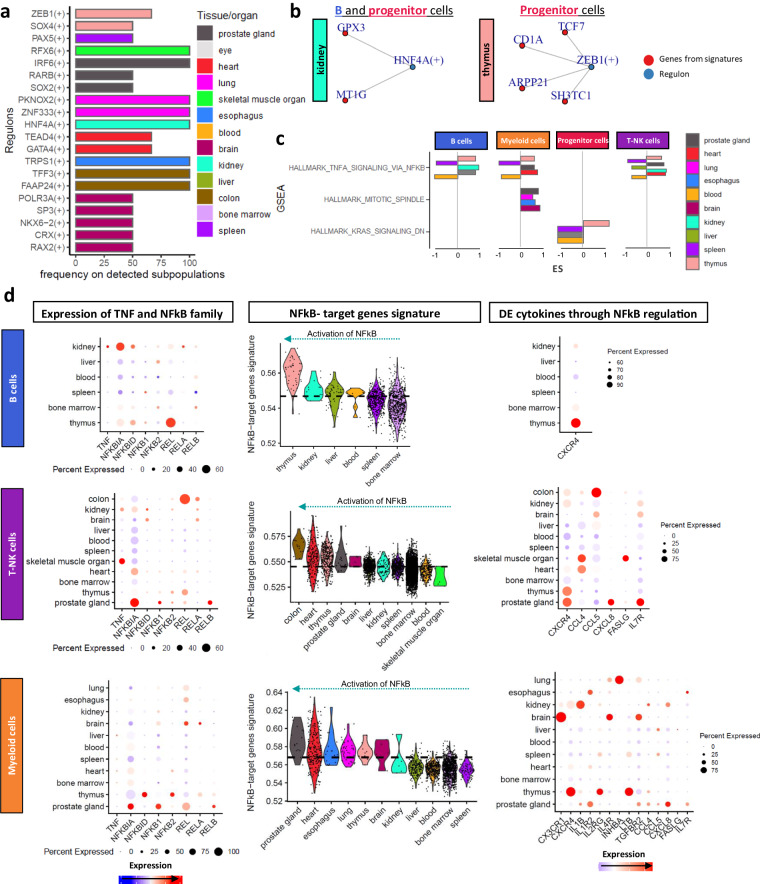
Functional analysis of differentially expressed genes across the different organs in each immune cell type. (**a**) Barplot showing the unique regulons in each organ (y axis) and the percentage of the detected immune cell subpopulations showing the regulons in each organ (x axis). (**b**) Network of the regulons (blue) and the genes associated with the gene-signature (red). (**c**) Barplot showing the shared pathways in the GSEA (y axis) per immune cell type and in each organ and the enrichment score values (x axis). (**d**) Analysis of the *TNFA* signaling pathway via *NFkB* in each immune cell type showing the expression of the *TNF* and *NFkB* family members, the target genes and the target cytokines.

T and NK (T-NK) cells emerged as the predominant immune cell types (n = 69,285, 60% of detected immune cells) and were divided into eight clusters expressing specific cell type makers, such as *CD8A*, *NKG7* and *FCGR3A* (Fig. 2a, Supplementary-Fig. 4a). Most cells (52.2%) were found in the naïve (n = 21,360) and memory (n = 14,784) clusters expressing *SELL* and were distinguished by the differential expression of activated genes such as *FOS* (Fig. 2b, Supplementary-Fig. 4a). Memory T cells were further divided into *CD4*+, *CD8*+ or *LEF1*+ clusters (Supplementary-Fig. 4b) with the last one being associated with thymus (hypergeometric distribution, p < 0.05) (Supplementary-Fig. 4c**)**. The *CD8*+ cells were found in spleen and liver, while the *CD4*+ in thymus, kidney, heart, prostate gland and bone marrow (hypergeometric distribution, p < 0.05) (Supplementary-Fig. 4c). Additionally, a Treg subcluster (n = 283) was found in the *CD4*+ subpopulation (Supplementary-Fig. 4d) displaying distinct transcriptomic signatures to each organ **(**Fig. 2c**)**. Τhymic Tregs (natural Tregs-nTregs) exhibited the highest levels of *FOXP3* and *CTLA4* expression, while splenic Tregs showed similar expression patterns with nTregs expressing *CTLA4*, *TNFRSF18* and *TIGIT* (Fig. 2c).

Five clusters associated with cytotoxicity (*CD56*+ NK, *CD16*+ NK, NK-T cells, NK like T cells and cytotoxic T cells), captured 47.5% of the defined T-NK cells. A cluster was characterized as *CD8*a/b (entry) cells and was fully linked with the thymus (225/227 cells) (hypergeometric distribution, p < 0.05) (Fig. 2b). Of the 69,285 T-NK cells, 46,733 (67.4%) were found in bone marrow, 7,949 (11.4%) in the liver, 5,197 (7.5%) in the spleen, 3,383 (4.8%) in the thymus, 2,253 (3.2%) in blood and 2,247 (3.2%) in the heart (Fig. 2b). Bone marrow and blood were associated with naïve and memory T cells (hypergeometric distribution, p < 0.05). Spleen and liver, on the other hand, were associated with the five cytotoxic clusters (Fig. 2b). The residual organs were accompanied by cytotoxic T-NK cells. T-NK cells expression profiles across organs showed high variability (Pearson’s-r: min = 0.77, max = 0.99) (Supplementary-Fig. 4e). The best correlation was observed between the liver and spleen (Pearson’s-r: 0.99) since they were associated with cytotoxic clusters and the most distinct organ was skeletal muscle (Pearson’s-r: 0.77–0.94).

A total of 21,800 myeloid cells were classified into ten clusters using known markers such as *CD14*, *FCGR3A*, *RETN*, *ITM2C* (Supplementary-Fig. 5a), including four transcriptionally distinct macrophage subpopulations (Fig. 2a). Among the cells, 60% were identified as *CD*14+, followed by 13.4% characterized as macrophages, 11.8% as *CD16*+, and 8.7% as dendritic cells (DCs). We captured platelets (n = 625, 2.8%) with blood and spleen, plasmacytoid DCs (n = 348, 1.5%) and neutrophils (n = 312, 1.4%) associated with bone marrow (hypergeometric distribution, p-value < 0.05) (Fig. 2b). Macrophages were found in spleen, lung and brain (hypergeometric distribution, p < 0.05) (Fig. 2b). Among the myeloid cells, 50.1% originated from the bone marrow (n = 10,926), with the remaining distribution as follows: 20.4% from blood (n = 4,460) and 9.1% from the liver (n = 1,993) (Fig. 2b). High heterogeneity was observed across the different organs in myeloid cells (Pearson´s r: 0.07–0.97) (Supplementary-Fig. 5b). The most distinct organs were the lung (r: 0.2–0.59) and the esophagus (r: 0.07–0.53) and the highest correlation was found between the spleen and liver (r = 0.99).

In the analysis of 2,933 macrophages, four clusters were identified comprising two clusters derived from the *CD14*+ lineage and two distinct subpopulations identified as alveolar and *RBPJ*+ macrophages, discerned through trajectory analysis (Supplementary-Fig. 5c). The highest number of macrophages was found in heart (n = 1,755, 59.8%), followed by lung (n = 463, 15.7%) and liver (n = 203, 6.9%). Using gene markers such as *TREM2*, *RBPJ*, *PPARG* and *FCN1* (Supplementary-Fig. 5d), we identified M1- and M2-associated clusters. The inflammatory *FCN1*+ _*S100A9/8* macrophages (n = 986, 33.6%) constituted the predominant subpopulation, mainly located in the heart, where we also identified *TREM2*+ macrophages (Fig. 2d). M2 polarization markers were found in *FOLR2*+ (n = 329, 11.2%), *RBPJ*+ (n = 527, 17.9%) and alveolar (n = 410, 13.9%) macrophages. Interestingly, *IgG*+ macrophage subcluster expressing *IGHG* genes without the presence of *CD79A* expression was observed in cells derived from the liver, spleen, blood, and a few from the bone marrow (Supplementary-Fig. 5e).

### Organ- and immune- related expression of genes by differential expression analysis across the different organs

To gain insight into the organ-specificity of the resident immune cells, we developed a strategy to highlight the organ-specific expression of genes (Methods). The identified organ-specific genes exhibited elevated expression levels compared to those in other organs. Although no single gene exhibited an exclusive reduction in expression specific to a particular organ, an overall downregulation of genes was observed.

In the context of B cells, seven signature genes were uniquely overexpressed in specific organs (*DNTT*, *DONSON*, *ACVR2B* and *RGS1* in thymus, and *MT1G* and *GPX3* in kidney) (Fig. 3b). In the T-NK cell analysis six genes (*PHGR1*, *SOX4*, *ACVR2B*, *DNTT*, *MTND1P23*, *GPX3*) exhibited distinctive expression patterns in specific organs (Fig. 3b). Specifically, *PHGR1* was expressed in the colon, *SOX4*, *ACVR2B* and *DNTT* in thymus, *MTND1P23* in brain and *GPX3* in kidney.

Focusing on the myeloid cells, 16 signature genes were found for thymus (*PRMT9, PPP1R14B, DONSON, ACVR2B, LILRA4, LINC00996, GZMB, DNTT, SMPD3, TPM2, PTCRA, SPIB, DNASE1L3, TSPAN13, TRAF4, LY9*). However, it is noteworthy noting that most of these genes were associated with the biology of plasmacytoid DCs, the sole subpopulation detected within thymic myeloid cells. Also, six signature genes were identified for the lung (*KLHL29, SLC19A3, STAC, PPARG, CYP27A1, FN1*), specifically linked to the biology of lung macrophages. Furthermore, two signature genes were found for the brain (FTX, C3), and one for the kidney (GPX3) (*GPX3*) (Fig. 3a).

In terms of the progenitor cells (lymphoid/ myeloid lineages), blood showed eight uniquely expressed genes (*CDA, TLR2, QPCT, CREB5, VCAN, CYP1B1, FCGR1A, MEGF9*) followed by 15 genes in thymus (*PTCRA*, *MIR181A1HG*, *SCAI*, *AEBP1*, *DNTT*, *ACVR2B*, *TCF7*, *CYP2U1*, *CD1E*, *CD1B*, *ELOVL4*, *CD1A*, *ARPP21*, *CD1C*, *SH3TC1*) and five genes (*APOE*, *GPX3*, *FTLP3*, *HSPA1B*, *MT1G*) in the kidney (Fig. 3b**)**. Since the thymus is involved in a specific maturation process, its signature genes were associated with T cell differentiation, such as *CD1E* (T cell marker). A detailed breakdown of signatures across various organs and immune cell types, along with insights into the anticipated or unexpected gene expressions, is presented in Table 2.

**Table 2:** The organs’ gene signatures in each immune cell type adding the etiology of the detected expression as microenvironmental effect/organ-specificity or influenced by the over-presented immune cell type. We defined as microenvironmental effects the expression of genes that are typically expressed in a particular organ and are not related to the immune cell type and as over-presented immune cell type etiology the genes that are typically expressed in a particular immune cell type. The genes that are not related to the previous categories were classified as undetermined reasons.

Immune cell type	Etiology of gene expression	thymus	lung	brain	kidney	colon	blood	liver
myeloid cells	microenvironment/organ-specificity	*DONSON* *ACVR2B* *DNTT*		*FTX*	*GPX3*			
myeloid cells	over-presented immune cell type	*PPP1R14B* *LILRA4* *GZMB* *SMPD3* *TPM2* *PTCRA* *SPIB* *DNASE1L3* *TSPAN13* *TRAF4* *LY9*	*SLC19A* *PPARG* *CYP27A* *FN1*	*C3*				
myeloid cells	undetermined reason	*PRMT9* *LINC00996*	*KLHL29* *STAC*					
T-NK cells	microenvironment/ organ-specificity	*ACVR2B* *DNTT*		*MTND1P23*	*GPX3*	*PHGR1*		
T-NK cells	over-presented immune cell type	*SOX4*						
progenitor cells	microenvironment/ organ-specificity	*MIR181A1HG* *ACVR2B*			*GPX3* *HSPA1*			
progenitor cells	over-presented immune cell type	*PTCRA* *DNTT* *TCF7* *CYP2U1* *CD1E* *CD1B* *ELOVL4* *CD1A* *ARPP21* *CD1C* *SH3TC1*			*APOE*		*CDA* *TLR2* *QPCT* *CREB5* *VCAN* *CYP1B1* *FCGR1A* *MEGF9*	
progenitor cells	undetermined reason	*AEBP1* *SCAI*			*FTLP3* *BMT1G*			
B cells	microenvironment/ organ-specificity	*DNTT* *ACVR2B* *DONSON* *RGS1*			*GPX3* *MT1G*			

Signature genes are found in different immune compartments highlighting organ-specificity or the predominant immune cell type of each organ and were expressed in a high proportion of cells (Supplementary-Fig. 6). Among the identified signature genes within each immune cell subpopulation, some were exclusive to a specific immune cell subpopulation and organ, while others were shared across different immune cell subpopulations but exclusively expressed in one organ. Ten genes were present in more than 50% of the immune cell subtypes (B, T-NK, myeloid, progenitors) across all studied organs (Fig. 3b, Table 2). Notably, *GPX3* gene was identified as a signature gene for kidney and *DNTT* and *ACVR2B* were specific to for thymus across myeloid, progenitor, B, and T-NK cells. Signature genes exclusive to specific organs, such as *CYP27A1, FN1, KLHL29, PPARG, STAC, SLC19A3* for the lung and *PHGR1* for the colon, were detected. However, these were found in a single immune subtype, with the colon associated only with T-NK cells and the lung linked solely to myeloid cells. The identified genes suggest either a microenvironmental effect (*e.g*., *DNTT* in thymus, *GXP3* in kidney) or an expected observation based on the overrepresented immune cell type in one organ (*e.g., PPARG* in macrophages derived from lung since it is a well-known marker for alveolar macrophages) (Table 2).

To validate our proposed signature genes for the thymus and kidney which were consistently expressed across immune cell types (Supplementary-Fig. 7a), we searched for additional confirmation from other publicly available datasets. Focusing on the kidney, we found expression of *GPX3* in three independent scRNA-seq datasets^11–13^ (Supplementary-Fig. 7b). Regarding the thymic signature, the expression levels of *DNTT* and *ACV2RB* were validated using bulk RNA-seq from sorted *CD19*+ thymic cells^14^ (Supplementary-Fig. 7c). Notably, no correlation was observed between doublet scores and the expression of these three genes in the associated tissues (DNTT in thymus: Pearson R = −0.18, ACVR2B in thymus: Pearson R = 0, GPX3 in kidney: Pearson R = 0.01).

### *In silico* functional analysis revealed transcription factor specificity and *NFkB* implication on localization

Going one step of organization further, we investigated the transcription factor (TF) activity (regulons) underlying expression pattern differences in different organs and immune compartments. The analysis was focused on the regulons that were uniquely found in one organ while being expressed across various immune cell subpopulations underlying the organ-specificity (Fig. 4a). Interestingly, eight regulons demonstrated organ-specificity and were found in all the defined subpopulations, *e.g., HNF4A* for the kidney and *TFF3* for the colon. Focusing on the regulons that appeared in more than three different organs, we found *PARG, POU2AF1, HOXB2* and *ESR2* (Supplementary-Fig. 8a). To establish connections between organ-specific signature genes and regulons, (Fig. 3b) we identified four regulons associated with nine signature genes (Table 3). Of these four, *HNF4* and *ZEB1* emerged as key regulators, impacting more than one gene (Fig. 4b). *HNF4* was associated with *GPX3* and *MT1G* in B and progenitor cells from kidney and *ZEB1* with *TCF4*, *CD1A*, *ARP21* and *SH3TC1* in thymic progenitor cells.

**Table 3:** Organ-specific TFs in relation to the genes of the defined signatures.

Regulons	Cell type	Organ	Gene in signature
*CRX*	myeloid_cells	brain	*C3*
*HNF4A*	myeloid_cells	kidney	*GPX3*
*TFF3*	T-NK_cells	colon	*PHGR1*
*HNF4A*	T-NK_cells	kidney	*GPX3*
*ZEB1*	progenitor_cells	thymus	*TCF7*
*ZEB1*	progenitor_cells	thymus	*CD1A*
*ZEB1*	progenitor_cells	thymus	*ARPP21*
*ZEB1*	progenitor_cells	thymus	*SH3TC1*
*HNF4A*	progenitor_cells	kidney	*GPX3*
*HNF4A*	progenitor_cells	kidney	*MT1G*
*HNF4A*	B_cells	kidney	*MT1G*
*HNF4A*	B_cells	kidney	*GPX3*

Using DE genes across immune subtypes and organs, GSEA (FDR < 0.01) identified 12/26 uniquely enriched pathways in different organs (Supplementary-Fig. 8b). Established pathways such as WNT signaling pathway in thymic progenitor cells^15^ and ‘allograft rejection’ in blood B cells^16^ serving as a validation for the analysis. Metabolic pathways were deregulated in immune compartments, particularly in the myeloid, with hypoxia being the most frequently affected across organs (spleen, heart, and kidney) (Supplementary-Fig. 8b). *TNF-α* signaling via *NFkB*, mitotic spindle, and *KRAS* signaling were targeted in more than three organs (Fig. 4c).

*TNF-α* signaling via *NFkB* exhibited activity in T-NK, myeloid and B cells. *TNFA* and *NFkB* family members expressions varied across organs and immune cell types (Fig. 4d). Specifically, *REL* was highly expressed in B and T-NK cells in the thymus, while *NFKBID* and *NFKB2* were expressed in the myeloid compartment. T cells from colon, myeloid cells from prostate gland and B cells from the thymus showed higher *NFkB* target genes activation compared to the other organs from the same immune cell compartment (Fig. 4d). The *NFkB* target gene activation in the maturation stage^17^ is expected, however, we observed additional activation of memory and mature memory B cells from thymus compared to spleen which in turn showed association with mature cells. To validate the observed *NFKB* activation in thymic B cells, we utilized the same data source, confirming the thymic gene signature through bulk RNA-seq analysis of sorted *CD19*+ thymic cells^14^. Our approach included both positive controls (BCR signaling pathway) and negative controls (T cell markers not expressed in B cells) (Supplementary-Fig. 8c). This validation reinforces the robustness of our findings, affirming the active involvement of the *NFKB* pathway in thymic *CD19*+ B cells (adjustment p-value = 4.52e-15 compared to T cell markers). In terms of immune cell types, macrophages exhibited the highest *NFkB* activation (Supplementary-Fig. 8d).

To further investigate the role of *NFkB* in cytokine activation and immune cell positioning^18,19^, we checked for DE target cytokines across different organs and immune compartments (Fig. 4d). A distinct set of cytokines was found in T-NK cells (*CCL4*, *CCL5*, *CXCL8*, *CXCR4*, *FASLG*, *IL7R*) and myeloid cells (*CX3CR1*, *CXCR4*, *IL1B*, *IL1R2*, *IL2RG*, *IL4R*, *INHBA*, *LTB*, *TGFBR2*) combined with different expression profiles across organs. *CXCR4* was the only cytokine overlapping between different immune compartment, particularly associated with thymic immune cells. Furthermore, specific cytokines were linked to distinct organs *e.g., IL7R* in T-NK cells from the prostate gland and *CX3CR1* in T-NK cells from the brain.

To validate the significance of cytokines in intercellular communication, we examined cell-cell interactions in thymic B cells and cardiac T-NK cells, both displaying robust *NFKB* pathway activity. *CXCR4-MIF* and *CXCR4-TGFB1* (Supplementary-Fig. 8e) was found shared between thymic B cells and T cells, while T-NK cells exhibited interactions with and myeloid cells through *CCL5-CCR1*, *CCL5-CCRL2*, and *CLL4-CCR1* was observed (Supplementary-Fig. 8f).

## Discussion

Recent studies have provided organ-atlases combined with in-depth characterization of cells but lacked biological relevance. In response, we introduce an immune cell meta-atlas using single-cell expression data from multiple organs from 12 publicly available projects in healthy donors (1/12 healthy donors from a liver transplantation). Our approach aligns with open science principles, promoting data reusability and contributing to a more collaborative research landscape. Compared to previous studies^5–8^, our analysis extends knowledge beyond the annotation of rare subpopulations focusing on immune cell residency in different organs. We aimed to investigate the changes that immune cells undergo while residing in host organs, their use of components of the host organ, and their potential roles beyond their defensive function.

Following previous publications^5,20^, our multi-organ immune cell map accurately delineated distinct immune subpopulations. This emphasizes that the results are reliable and can be reproduced when combining information from different sources. Additionally, our findings reveal the existence of organ-specific macrophage populations, aligning with earlier studies^7^. The transcriptomic profiles of Tregs found in various organs suggest that these cells have undergone local adaptation to maintain immune homeostasis. Interestingly, Tregs in the spleen appear to have a thymic origin, while Tregs in other organs suggest differentiation in the periphery, rather than being thymic generation^21^. Additionally, we uncovered two clusters of monocyte-dependent macrophages and another two distinct clusters possibly originating from the yolk sac stage like many organ-resident macrophages^22,23^. Remarkably, the detected *IgG*+ macrophages, are known to be organ-resident^24^ and implicated in tumor-associated inflammation^25^.

In lymphatic organs, both primary and secondary, the expected distribution of immune cell types was evident, while non-lymphoid organs exhibited an association with myeloid cells. Interestingly, lung and esophagus displayed non-correlated patterns due to their relationship with specific subtypes of macrophages. We observed a novel correlation between immune cells from the thymus and kidney, with novel correlation across different immune compartments. We found that progenitor cells from the kidney were clustered with classical thymocytes, indicating a thymocyte-like phenotype. This relationship is intriguing given the previous association between thymus and kidney diseases, although the underlying mechanism remains unknown^26–28^. Additionally, our findings provide compelling evidence indicating that thymic B cells may indeed originate from the bone marrow^29^. Even though the immune cells are consistent across different organs, the upregulation and downregulation of genes observed in our study could be attributed to organ-specific gene expression programs. This emphasizes the necessity for immune cells to adapt to the specific microenvironment of each organ and carry out specialized functions.

In the reported organ-specific signatures of the B cell fraction, a noteworthy observation centered around the unique expression of the genes *RGS1* and *ACVR2B* in the thymus. These genes are implicated in cytokine signaling, providing further support for their significance in facilitating the homing of cells to diverse organs^30,31^. Additionally, the *GPX3* gene was uniquely expressed in kidney in line with previous publications showing its high expression in proximal tubules^32^. For T cells, the gene *PHGR1*, associated with the colon, exhibited exclusive expression in the gastrointestinal tract, as highlighted by a previous publication^33^.

Some of these upregulated genes were present in all the major immune cell types per organ indicating the potential reliance on specific factors or molecules present in the respective host organ microenvironment (*e.g., DNTT* and activin receptor*, ACVR2B*, in thymus, *GXP3* in kidney). Conversely, other genes with high organ-specificity were expressed exclusively in one cell type without direct association with the host organ highlighting an expected observation based on the prevalence of a particular cell type within a given organ (*e.g., SOX4* expression in thymus T cells came from thymocytes differentiation^34^, *PPARG* in macrophages derived from lung which is a marker for alveolar macrophages^35^). Focusing on the microenvironmental effect of host organs, the immature stage of B and T cells express *DNTT*, but the maturation events require its silencing^36,37^. Here, we report that a high percentage of cells within the thymus continue to express *DNTT* suggesting a potential association with microenvironmental signals within this organ. Kidney-derived immune cells express *GPX3*, an exclusive kidney antioxidant enzyme that protects high metabolic activity cells from oxidative stress, as shown in previous studies on macrophages during kidney disease^32,38^. The comprehensive validation across external cohorts enhances the robustness of our findings, mitigating potential bias introduced by variations in batches within our meta-atlas. In addressing potential non-biological factors, our comparison of gene expression with the doublet score revealed no correlation. This underscores the data’s remarkable reusability, reinforcing its capacity to unveil novel biological insights. In relation to the *GPX3* gene, we found that *HNF4A*, a transcription factor associated with kidney pathophysiology^39^ serves as its regulator.

Our results showed deregulation of metabolic pathways which supports that immune cells altered them to be able to enter and adapt to the environment of the host organ contributing to homeostatic tissue function^40^. *TNFa* signaling via *NFkB* pathway emerged as the most targeted pathway showing varied *NFkB* activity through distinct expression patterns of *NFkB* family members and diverse expression profiles of the target genes in various organs. The *NFkB* signaling pathway is involved in normal cellular functions such as inflammation and immune cell interactions^41,42^, however in our meta-atlas the data originated from healthy organs. We observed an activation of the *NFkB* target genes in specific organs where such immune cells are not commonly found (*e.g*., B cells in thymus) supporting a potentially critical role of *NFkB* in immune cell homing. We validated the activation of *NFkB* target genes in thymic B cells using an external dataset. This validation ensures the robustness and reproducibility of our results across various experimental designs, as the dataset involved FACS-sorted *CD19*+ B cells and bulk RNAseq. Previous publications showed the implication of *NFkB* in localization since it regulates migratory dendritic cells^43^ and new T cells, and recent thymic emigrants up-regulated *IL-7Rα* once they leave the thymus which is a target gene of the *NFkB* transcription network^44^. Another recent study proposed that *NFkB* signaling has a specific role in tissue-resident memory in *CD8*+ T^45^ cells further supporting our results of the implication of *NFkB* activation on cell positioning. Moreover, our study revealed that macrophages exhibit the highest *NFkB* activation, aligning with prior research suggesting that *c-Rel* has distinct effects on gene expression, contingent upon whether macrophages are tissue-resident or elicited from the blood^46^. Knowing its role in the transcriptional activation of cytokines, we found different repertoires of cytokines through *NFkB* regulation in different organs further supporting the role of *NFkB* in cell positioning through cytokines^18^.

Taken together, this study reports organ-specific genes expression patterns in immune cells due to the microenvironment of the host organs. By identifying both unique and shared regulons and pathways, we underscore the functional impact of altered expression profiles as immune cells traverse different organs. The most remarkable finding of this meta-atlas analysis demonstrated the implication of the *NFkB* pathway in cellular positioning. Alterations in gene expression and metabolic pathways implying immune cells are not only involved in defending host organs but also adopt the host environment and contribute to their homeostasis and repair. The ability of immune cells to sense and respond to changes in the environment of different organs further highlights the importance of understanding the interplay between immune cells and host organs in health and disease.

## Methods

### Data collection and aggregation

162 loom files were downloaded from the 12 different projects within the HCA consortium (Supplementary Table 1). In more detail, we focused on 14 different organs: prostate gland^7,47^, eye^48^, heart^7,49^, skeletal muscle organ^7,50^, blood^20^, liver^20^, spleen^20^, brain^1,51^, kidney^12^, colon^52^, esophagus^7^, lung^7^, thymus^53^, bone marrow (1 M Immune Cells project, https://www.ebi.ac.uk/gxa/sc/experiments/E-HCAD-4/downloads). Of note, one project involved healthy donors from liver transplantation including samples from liver, blood and spleen (Supplementary Table 1). To facilitate downstream analysis, the loom files were transformed into Seurat objects with the function as.Seurat() from *Seurat*^54^ package in R. After incorporating metadata information, each object was screened for immune cells based on the expression of the *CD45* immune marker. Specifically, cells were considered immune cells if they exhibited at least one read of the *PTPRC* - *CD45* surface marker gene. This screening process led to a reduction of immune cells within each file, particularly in the case of bone marrow, where up to 80% of cells were excluded, primarily from the progenitor lineage. The individual files were merged into a single file. The full script of the process is available at GitHub (https://github.com/MariaTsayo/mulTI_Metatlas/) and under the DOI of Zenodo (10.5281/zenodo.10469288).

### Quality control, batch effect correction and clustering

We performed all downstream pre-processing with Seurat^54^. For quality control, we excluded cell barcodes with <1,000 Unique Molecular Identifiers (UMI), <200 detected genes, or mitochondrial expression >15%. In addition, we excluded genes detected in < = 4 cells. Next, we applied the functions NormalizeData(), FindVariableFeatures(), ScaleData() and RunPCA() of Seurat (with default parameters). Batch effect correction was performed with Harmony^55^ using the top 30 Principal Components (PC) as input. We considered as batches the cells coming from (i) the same sample and (ii) the same subproject of the HCA consortium. The validation of the data integration was performed visually with UMAP using the function RunUMAP() on the first 30 PCs and quantitatively with the Local Inverse Simpson’s Index using the *LISI* package in R (Supplementary-Fig. 9a).

To cluster cells into groups, we used the function FindNeighbors(reduction = “harmony”, 30 PCs) and then determined the clusters based on the function FindClusters(). Low-quality clusters (*e.g*., high expression of mitochondrial or rRNA, doublets) were removed from the downstream analysis. Moreover, doublets score was calculated using scDblFinder package in R. The characterization of the cells was performed by subsetting and reapplying FindNeighbors(reduction = “harmony”, 30 PCs) and FindClusters().

### Downstream analysis

Cell annotation was performed manually using the most significant markers in each cluster as well as comparing the results with *SingleR* and *EnrichR* tools in R. A publicly available annotation of the tonsil was used for in-depth characterization of the immune applying *Azimuth*^9^. Differential expression analysis was performed considering as a threshold the presence of at least 40 cells in each analyzed group. Except for the default parameters of FindMarkers() we used as an additional criterion the minimum percentage of cells expressing one gene to be greater than 50% on the group of interest helping to avoid the detection of genes that were expressed in only a small proportion of cells. A pseudobulk table was generated using the function AverageExpression() from the Seurat package. The *UCell* package^56^ was used to calculate the expression gene signature of *NFkB* target genes per cell using the AddModuleScore_UCell() function. To score the signatures in the bulk RNAseq data, we utilized the Single Sample Gene Set Enrichment Analysis (ssGSEA) from the *GSVA* package in R. Gene set enrichment analysis (GSEA) for each gene list was performed using the database of MsigDB under the package *clusterProfiler*^57^ in R. To infer transcription factor (TF) activity (known as regulons), we used *pySCENIC* on the scRNA-seq raw matrices from the different organs and immune cell types. The regulon activity per cell was quantified using an enrichment score for the targets of each regulon (AUCell). Additionally, a regulon specificity score (RSS) was computed using the Jensen-Shannon Divergence^58^. Pseudotime analysis was performed using *slingshot* and cell-cell communication using *LIANA* packages in R. For visualization we used the *Seurat*, *ggplot2*, *ComplexHeatmap* and/or *corrplot* packages in R.

### Detection of organ-specific gene signature

To explore the organ-specificity of immune cells, we aimed to identify distinct gene signatures by setting a threshold of a minimum of 40 immune cells per comparison. Differential expression analysis was conducted for each organ against all others within each immune cell type category using FindMarkers(), with a criterion of at least 50% of cells expressing a gene within the group of interest. Uniquely overexpressed genes were then screened through hierarchical cluster analysis using the pseudobulk table generated by the AverageExpression() function. The resulting gene sets, defined as organ-specific signatures, exhibited clear organ-specificity (Supplementary-Fig. 9b). This analysis was repeated considering down-regulated genes for each organ within each immune cell type.

### Supplementary information

Supplementary Information

Supplementary Table 1

## Data Availability

All loom files were downloaded from the HCA portal (https://data.humancellatlas.org/) (Supplementary Table 1). The scRNA-seq expression object as well as the metadata of the cells are available at Zenodo (10.5281/zenodo.10197112)^59^.

## Code availability

All the scripts for the analysis of the loom files up to the visualizations are available on Github (https://github.com/MariaTsayo/mulTI_Metatlas/) and under the DOI of Zenodo (10.5281/zenodo.10469288)^60^ with a detailed description of each one.
