# Supplementary material for: Multi-organ single-cell transcriptomics of immune cells uncovered organ-specific gene expression and functions
Source: Sci Data. 2024 Mar 27;11:316. doi: 10.1038/s41597-024-03152-z (PMC10973478; doi:10.1038/s41597-024-03152-z)
Supplement: Supplementary file 1 — Supplementary Information [file 41597_2024_3152_MOESM1_ESM.pdf]

Multi-organ single-cell transcriptomics of immune cells uncovered organ-specific gene expression and functions

Tsagiopoulou et al.

Supplementary information

Table of Contents

Figure S1 2

Figure S2 2

Figure S3 3

Figure S4 4

Figure S5 5

Figure S6 6

Figure S7 7

Figure S8 8

Figure S9 9

References 9

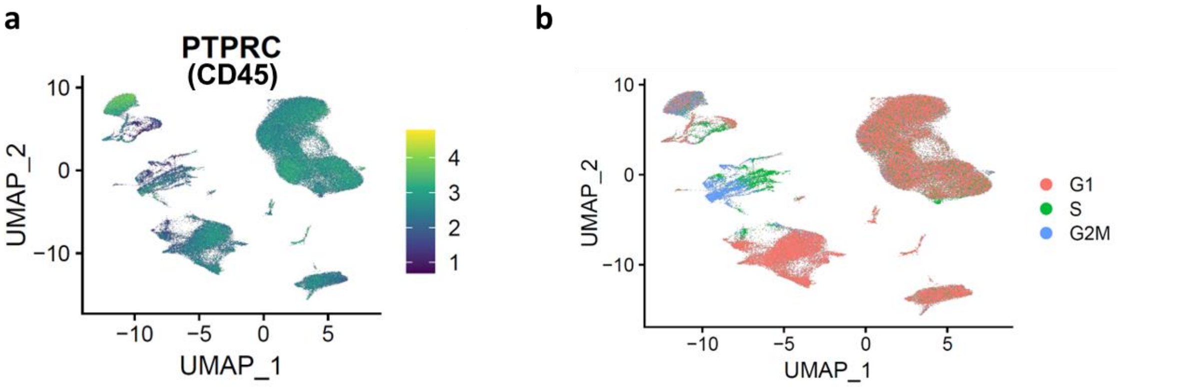

Figure S1. **a,** UMAP showing the expression levels of *CD45* through different immune compartments highlighting the lack of the non-thymic progenitor cells **b,** The UMAP plot of the immune cells coloured by the cell cycling markers.

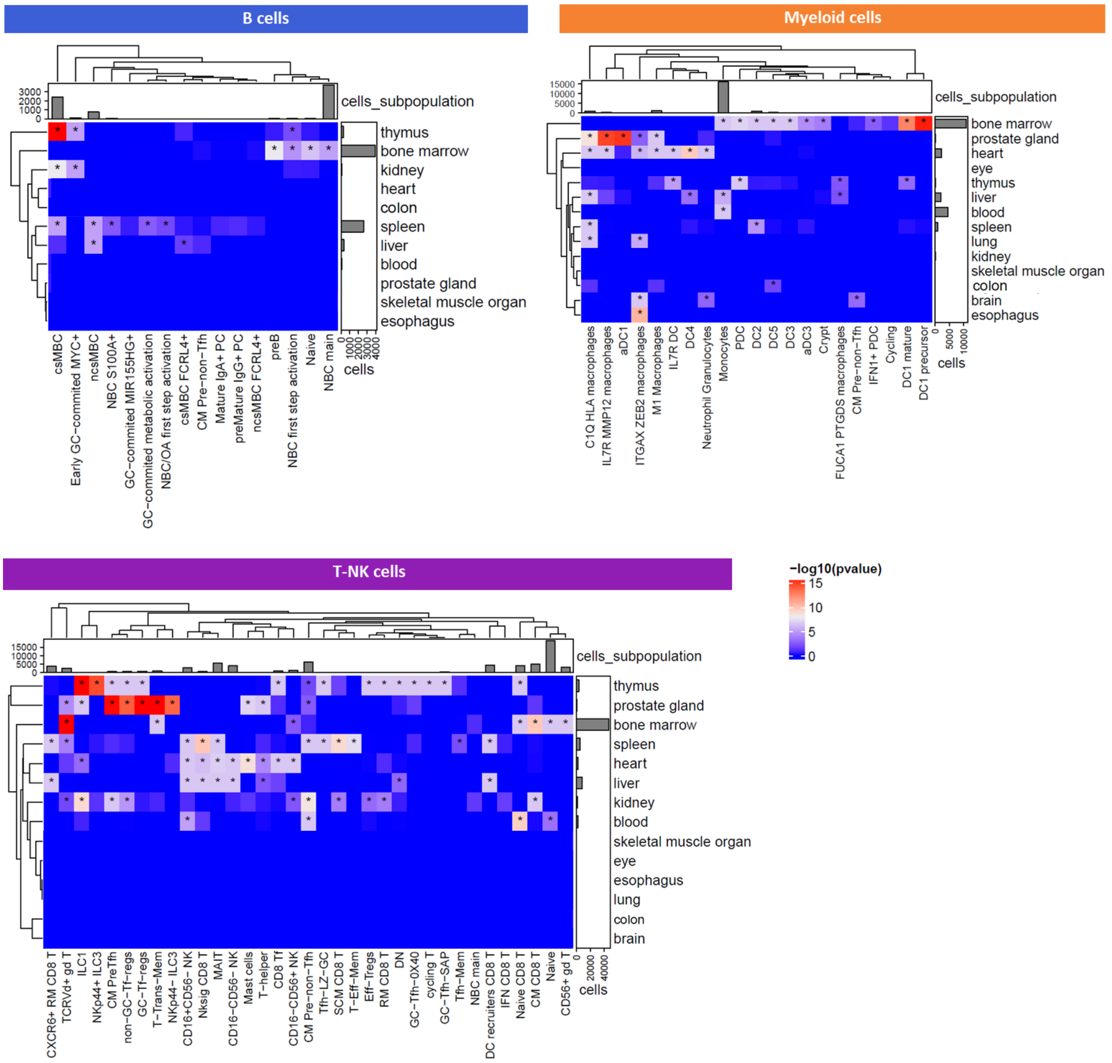

Figure S2. Heatmap showing the -log10(p-value) of the hypergeometric distribution between the annotation of tonsil atlas[1] and the different organs. The asterisks indicate statistically significant enrichment p-value<0.05 (hypergeometric distribution).

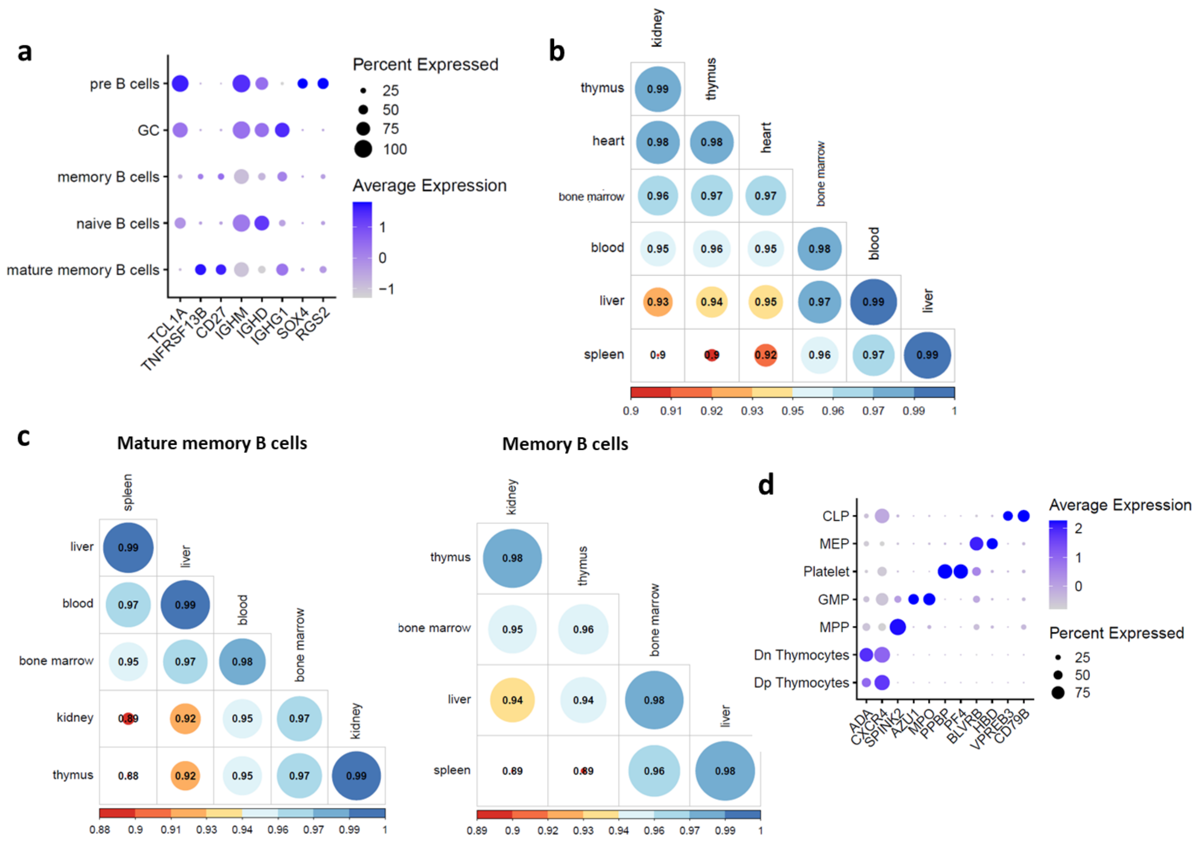

Figure S3. **a,** Expression levels and percentage of cells expressing the gene markers. **b,** Correlation (Pearson's r) plot between the expression profiles of B cells in different organs. **c,** Correlation plots between the expression profiles of mature memory (left) and memory B cells (right) subpopulation in different organs which include more than 10 cells. **d,** Expression levels and percentage of progenitor cells expressing the gene markers.

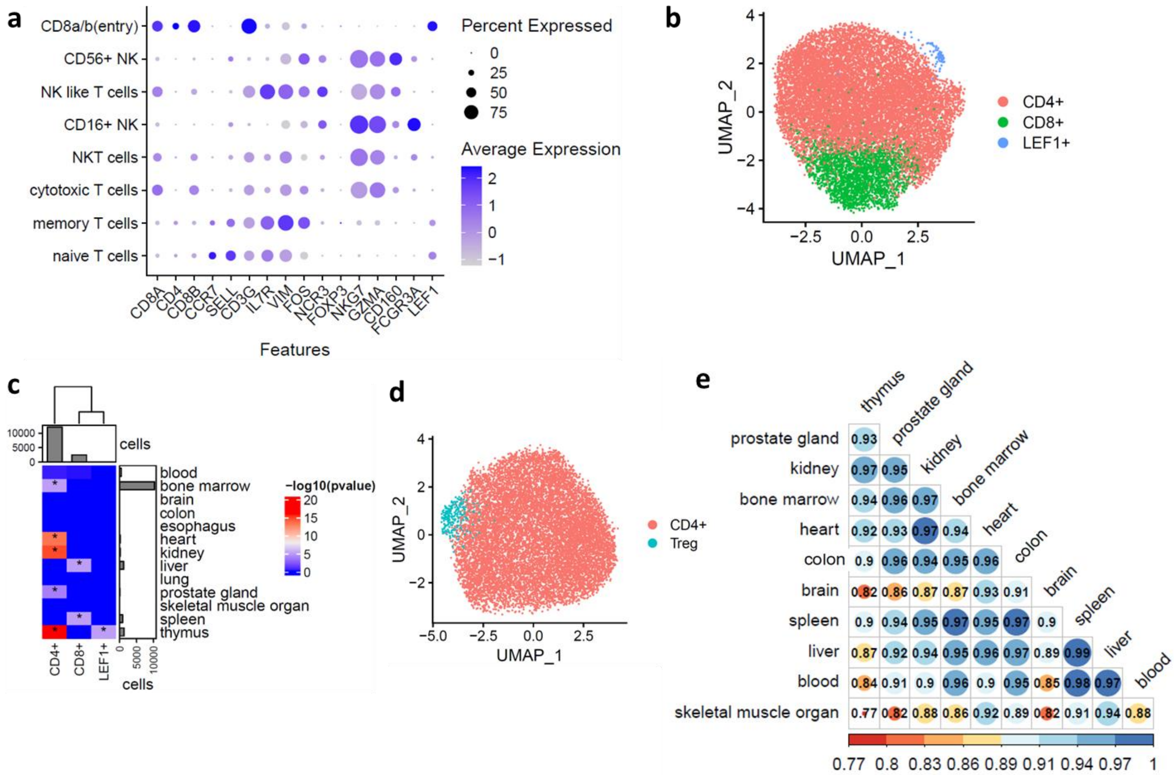

Figure S4. a, Expression levels and percentage of T-NK cells expressing the classical gene markers. **b,** The UMAP plot of the memory T cells. **c,** Heatmap showing the -log10(p-value) of hypergeometric distribution between memory T cell subpopulations and the different organs. The asterisks indicate statistically significant enrichment p-value<0.05 (hypergeometric distribution). **d,** The UMAP plot of the *CD4*+ memory B cells including Treg subpopulation. **e,** Correlation plot of the expression profiles of T-NK cells in different organs.

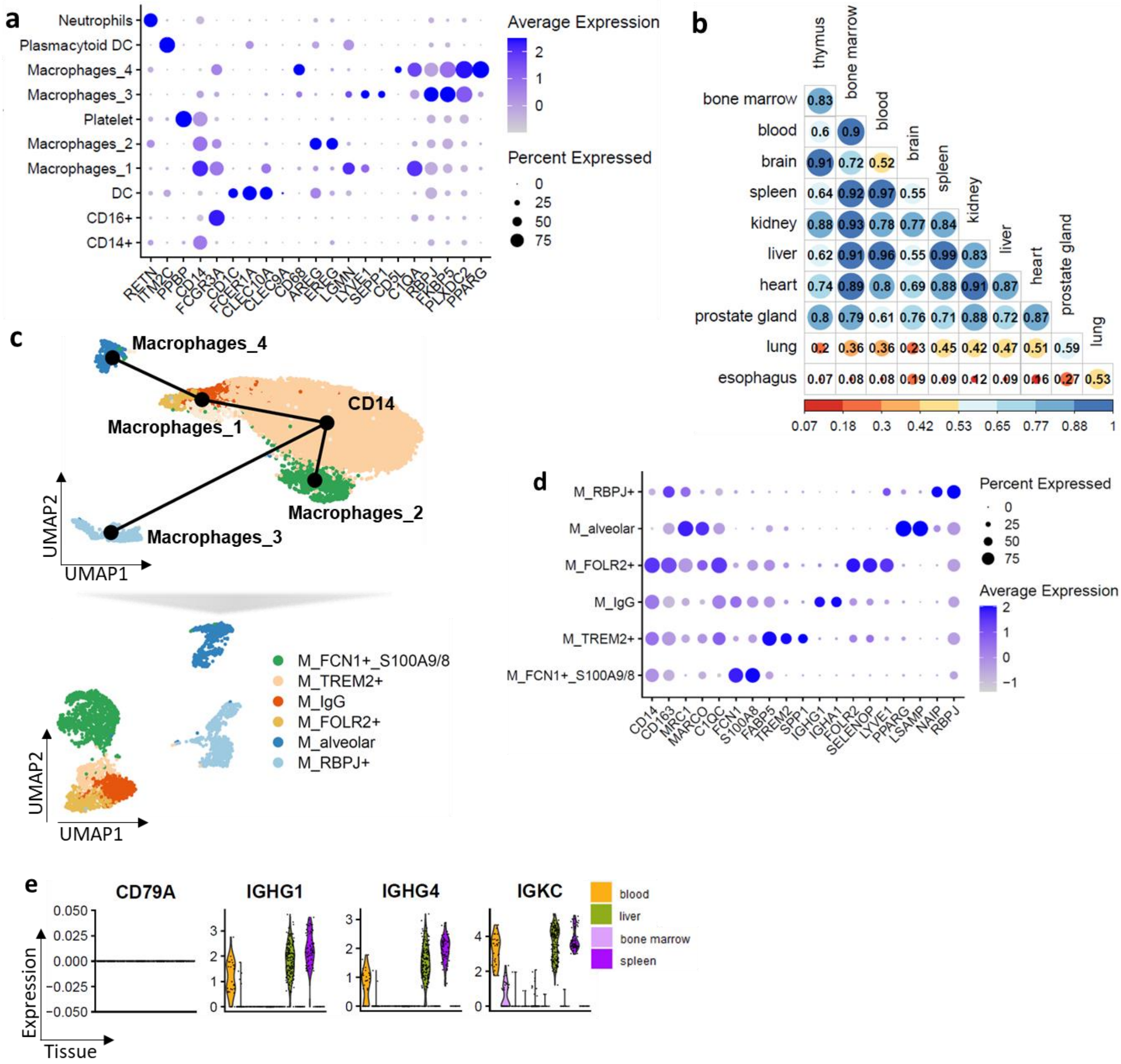

Figure S5. a, Expression levels and percentage of myeloid cells expressing the gene markers. **b,** Correlation plot of the expression profiles of myeloid cells in different organs. **c,** The UMAP plot of the macrophage subpopulations including the *CD14+* cells. The lines represent the trajectories using the slingshot package in R. Beyond, zoom-in UMAP plot of the characterized macrophage subpopulations. **d,** Expression levels and percentage of macrophages expressing the gene markers. **e,** Violin plots showing the expression of the *CD79A* marker of B cells as well as the expression of the IGHG genes in different organs.

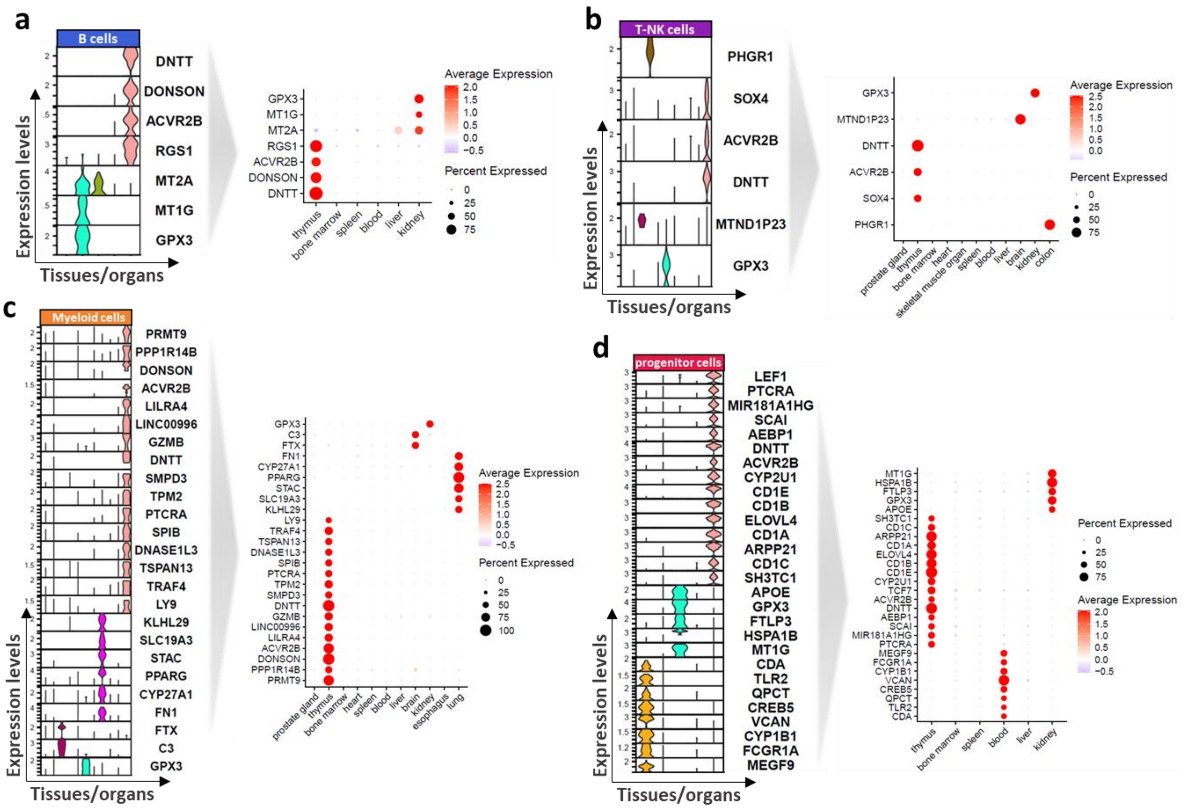

Figure S6. Violin plots for each immune subtype showing the expression of the gene signatures in each tissue and dotplots showing the expression levels and the percentage of cells in each tissue expressing the gene in **a,** B cells, **b,** T-NK cells, **c,** myeloid cells and **d,** progenitor cells.

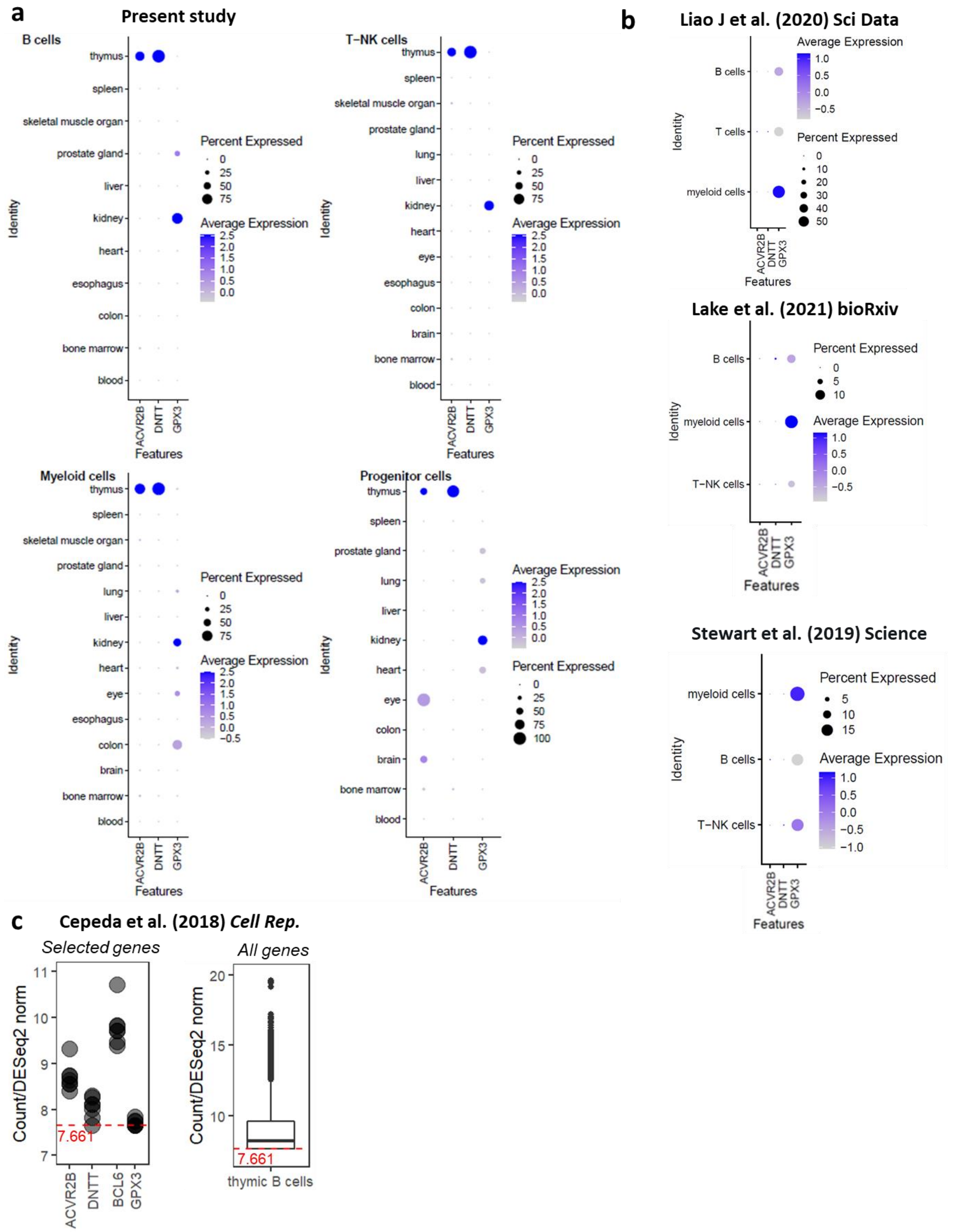

Figure S7. Validation of gene signatures using additional datasets. a, Expression levels and percentage of the immune cells expressing the *ACVR2B*, *DNTT*, *GPX3* genes in the present study. **b,** Expression levels and percentage of the immune cells expressing *GPX3* gene in three additional datasets serving as validation cohorts. **c,** The dotplot (left) shows the expression levels of *DNTT* and *ACV2RB* in bulk RNA-seq data from sorted *CD19*+ thymic cells. *BCL6* was used as a positive control gene and *GPX3* as a negative control. The boxplot (right) displays the expression levels of all genes after DESeq2 normalization. The red lines highlight the minimum level of expression of the DESeq2 normalization.

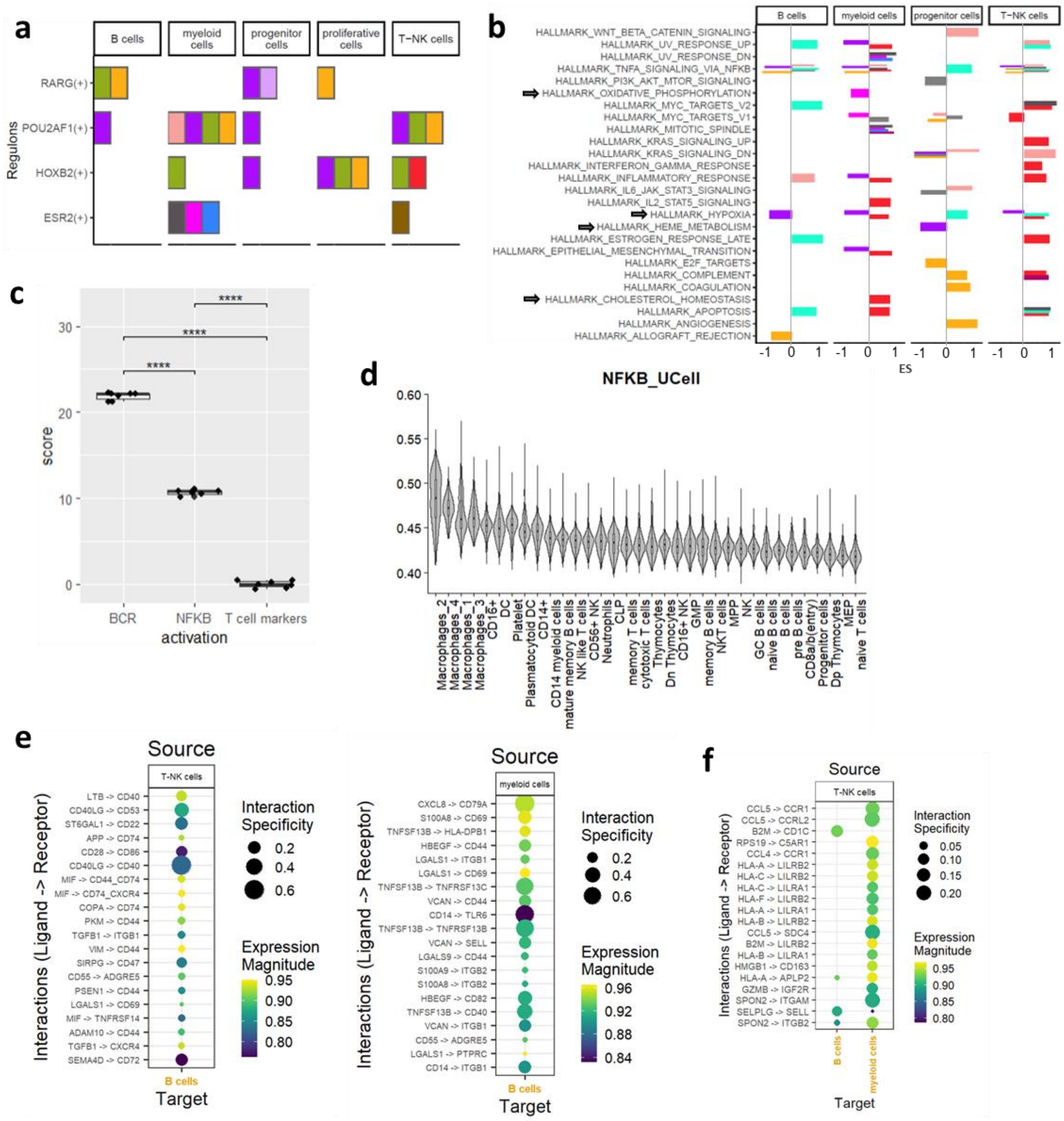

Figure S8. a, Barplot showing the shared regulons across organs (y axis) and in which immune cell subpopulations they were detected (x axis), colored by organ. **b,** Barplot showing the pathways that are enriched according to the GSEA (y axis) per immune cell type and in various organs and the ES values (x axis). Arrows highlighting the metabolic pathways. **c,** Boxplots presenting the NFkB target gene activation on *bulk RNA-seq data from sorted CD19+ thymic cells* compared to BCR pathway (Hallmark database) as well as to T cell markers (*CD3D*, *CD3E*, *CD4*, *CD8A*, *CD8B*). **d,** Violin plots showing the activation of NFkB targets genes in the different immune cell types including a deeper annotation of the cells. **e-f,** Cell-cell communication analysis showing the interaction specificity and the expression magnitude for **(e)** B cells (Receptor) with T-NK cells (ligand) and myeloid cells (ligand) in thymic cells **(f)** T-NK cells (ligand) with B cells (Receptor) and myeloid cells (Receptor) in cells from heart.

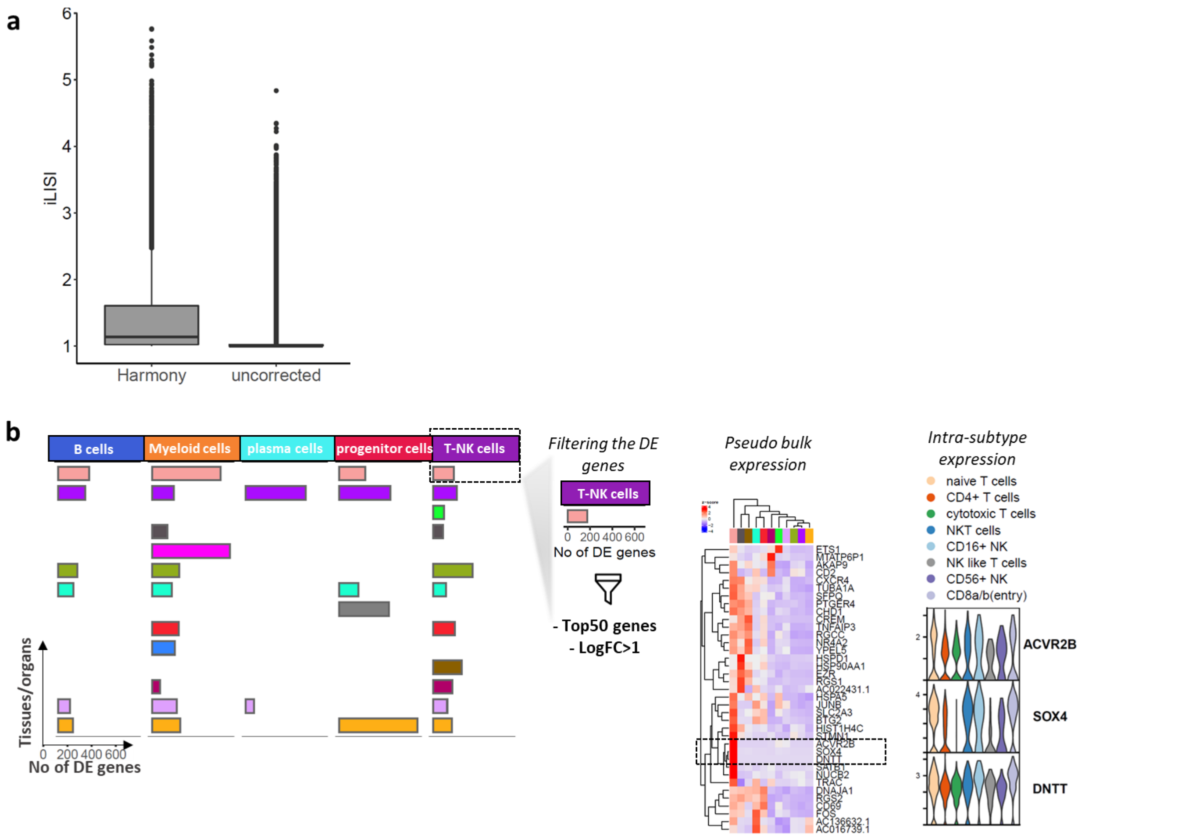

Figure S9. a, Boxplot showing the iLISI scores for uncorrected and corrected values after running Harmony concerning the different organs. **b,** Graph explaining the definition of the gene signature per organ in each immune cell type. The barplot shows the number of differentially expressed genes (overexpressed genes) in each organ splitting by the defined immune cell types. A filtering for the significantly overexpressed genes was applied and a hierarchical clustering analysis was performed using the top 50 genes. Finally, an intra-immune subtype expression analysis was performed checking the specificity of the expression.

References

1. Massoni-Badosa, R. *et al.* An Atlas of Cells in the Human Tonsil. *bioRxiv*, 2022.2006.2024.497299 (2022).
